# Supplementary material for: Global-scale control of extensional tectonics on CO2 earth degassing
Source: Nat Commun. 2018 Nov 2;9:4608. doi: 10.1038/s41467-018-07087-z (PMC6214998; doi:10.1038/s41467-018-07087-z)
Supplement: Supplementary file 1 — Supplementary Information [file 41467_2018_7087_MOESM1_ESM.pdf]

Global-scale control of extensional tectonics on CO_2 earth degassing

Giancarlo Tamburello[1]*, Silvia Pondrelli[1], Giovanni Chiodini[1], Dmitri Rouwet[1],

[1] *Istituto Nazionale di Geofisica e Vulcanologia, Sezione di Bologna, via Creti, 12, 40128, Bologna (Italy)*

* *corresponding author email: giancarlo.tamburello@ingv.it*

Supplementary Methods

Gas discharge coordinates and references

Geographic coordinates of the gas discharges that have been discussed in this work are shown in the Supplementary Data. Values are expressed in decimal longitude and latitude, displaying only the first decimal place to discourage the use of the dataset for smaller scale studies. We added for each point the corresponding information : country, altitude (extracted from the global digital elevation model GTOPO30, https://lta.cr.usgs.gov/GTOPO30), geology and era of the terrain (extracted from the general geologic map of the world[1]; https://mrdata.usgs.gov/geology/worldgeol.html), distance from the nearest fault and sliptype, distance from the nearest Holocene volcano. Here is a list of the countries hosting gas discharges with an updated reference (starting from the reference reported in Barnes et al.[2]): Afghanistan[2,3], Algeria[4], Argentina[6], Armenia[7], Australia[8], Austria[9,10], Azerbaijan[11,12], Azores[13], Bosnia and Herzegovina[15,16], Brazil[17], Bulgaria[18-23], Cameroon[24], Canada[25,26], China[27], Colombia[28], Croatia[29], Czech Republic[30-32], Ecuador[33], Ethiopia[34,35], France[36,37], Germany[38,39], Greece[40,41], Hungary[42-44], India[45-47], Indonesia: Java[48], Italy[49-53], Japan[54], Lesser Antilles[56], Macedonia[57], Mexico[58-60], Nepal[61], New Zealand[62], North and South Korea[63,64], Papua New Guinea[65], Perù[66-69], Philippines[70], Poland[71,72], Portugal[73], Romania[74-76], Russia: Kamchatka[77], Russia[78,79], Serbia[80,81], Slovakia[30-32], Slovenia[82], Spain[83], Sweden[84], Switzerlan[85], Taiwan[86,87], Tanzania[88], Tunisia[89], Turkey[90-92], Uganda[93], UK: Great Britain[94], Ukraine[64], USA[95-105], Vietnam[106].

Distance between gas discharges and nearest faults

The calculated distances between gas discharges and nearest faults show that the majority of the sites are near (<100 km) an active fault. In particular, this is true for normal and transcurrent faults (Supplementary Fig. 1).

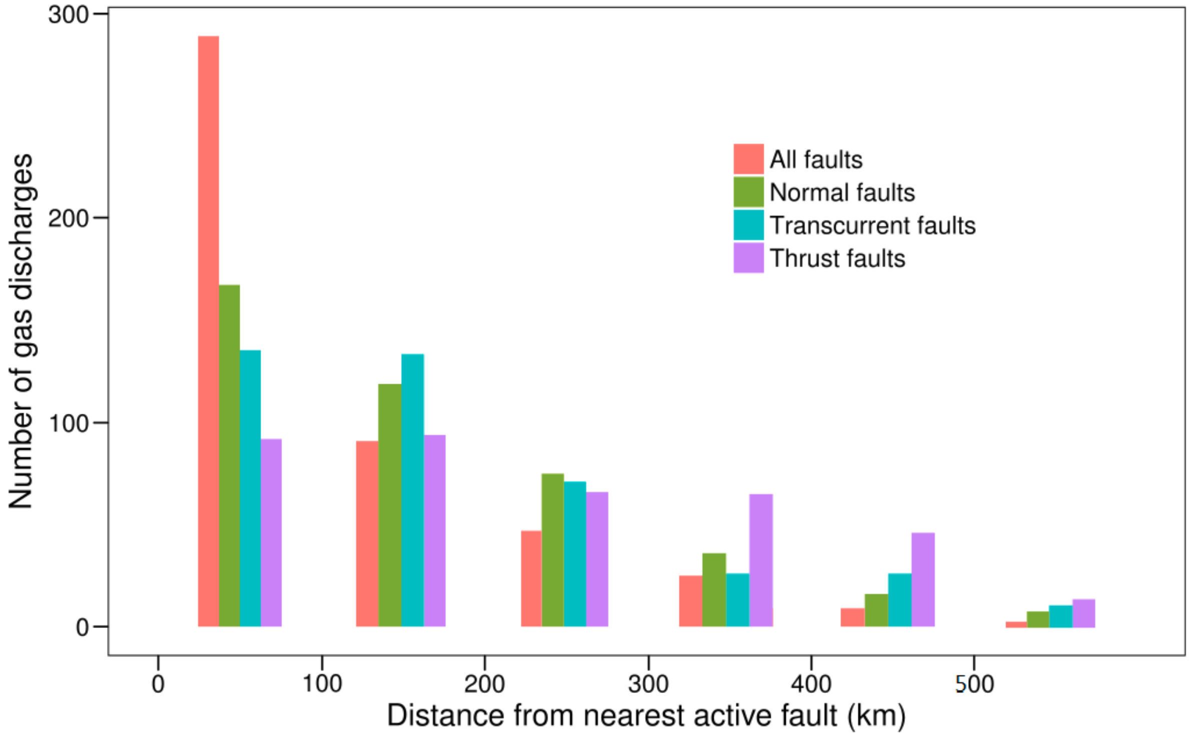

Supplementary Figure 1: Histogram of distances between gas discharges and nearest faults for different type of active faults. The bin width is set at 100 km.

Statistical distribution of count data

The hexagonal grids allowed to count the number of gas discharges and earthquakes for each cell. Faults are line features and therefore have been counted in terms of total length (in meters) for each cell.

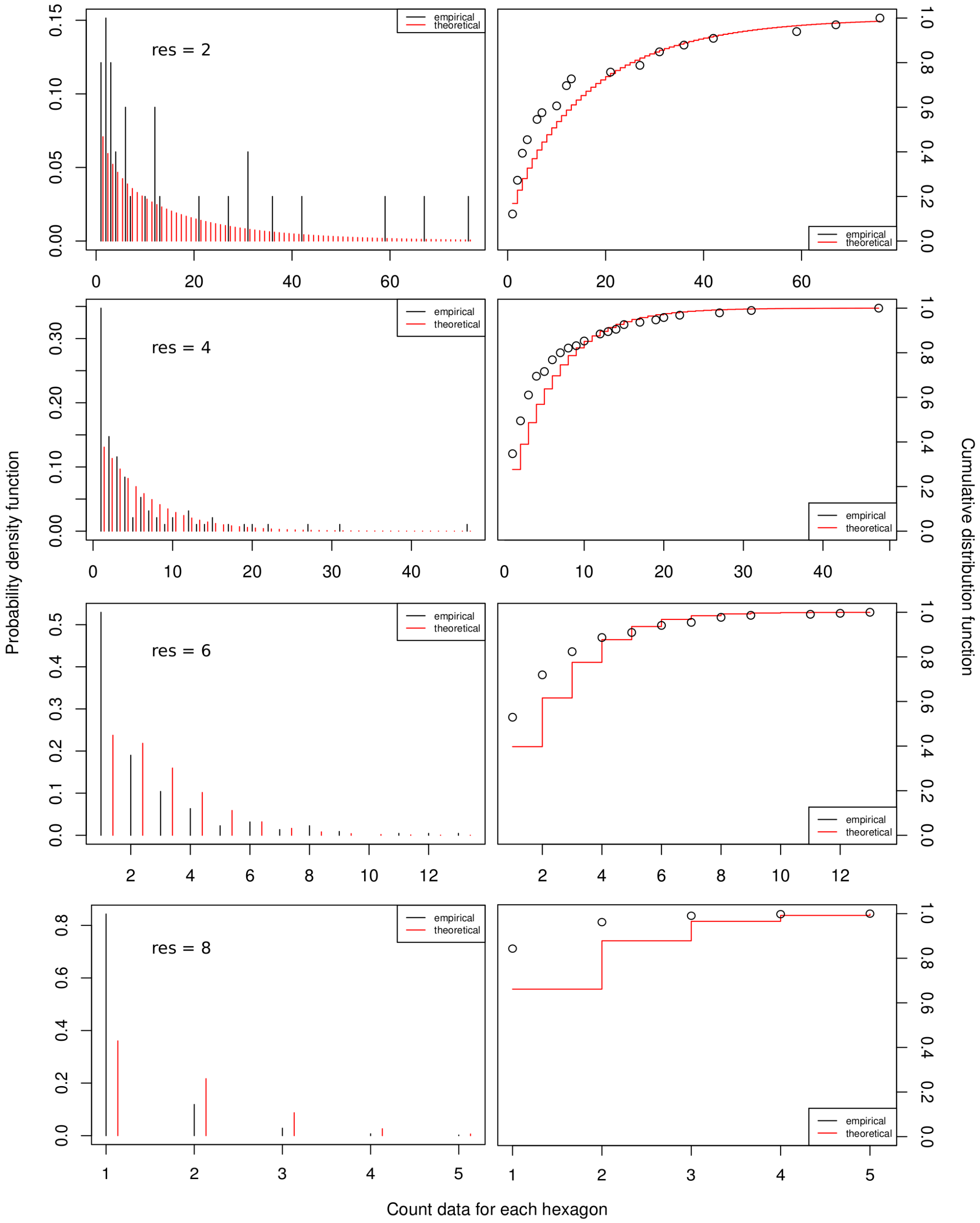

Supplementary Figure 2: Frequency histograms (left) and cumulative frequency graphs (right) for gas count data for hexagonal grids of different resolutions.

Count data are usually non-normally distributed and are treated as random variables. Supplementary Fig. 2 shows the frequency histograms and cumulative frequency graphs of

gas counts for grid resolutions ranging from 2 to 8. We calculated and overimposed the maximum-likelihood negative binomial fitting distribution.

Groundwater and gas discharges spatial distributions

Supplementary Fig. 3 shows "the global distribution of modern groundwater as a depth if it was extracted and pooled at the land surface like a flood"[107] and the mapped gas discharges. For the former we considered the calculated depths using the geomatic data, groundwater recharge and porosity as model input parameters (for further details refer to Gleeson et al.[107]).

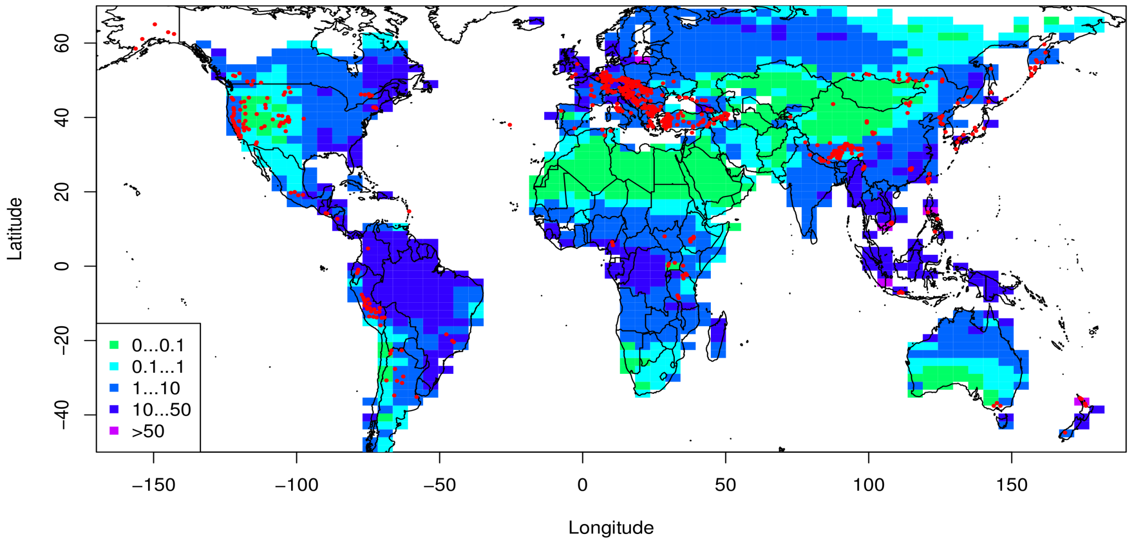

Supplementary Figure 3: Carbon dioxide discharges (red circles) and global distribution of modern groundwater as thickness (in meters) of extracted water from the aquifer and pooled at the land surface[107].

Global probability of compressional tectonic regimes

For completeness, the same statistical procedure described in Methods has been applied for compressional tectonic regimes. The result shows lower spatial correspondence between the main degassing regions and the areas with high probability of compressional tectonic (Supplementary Fig. 4).

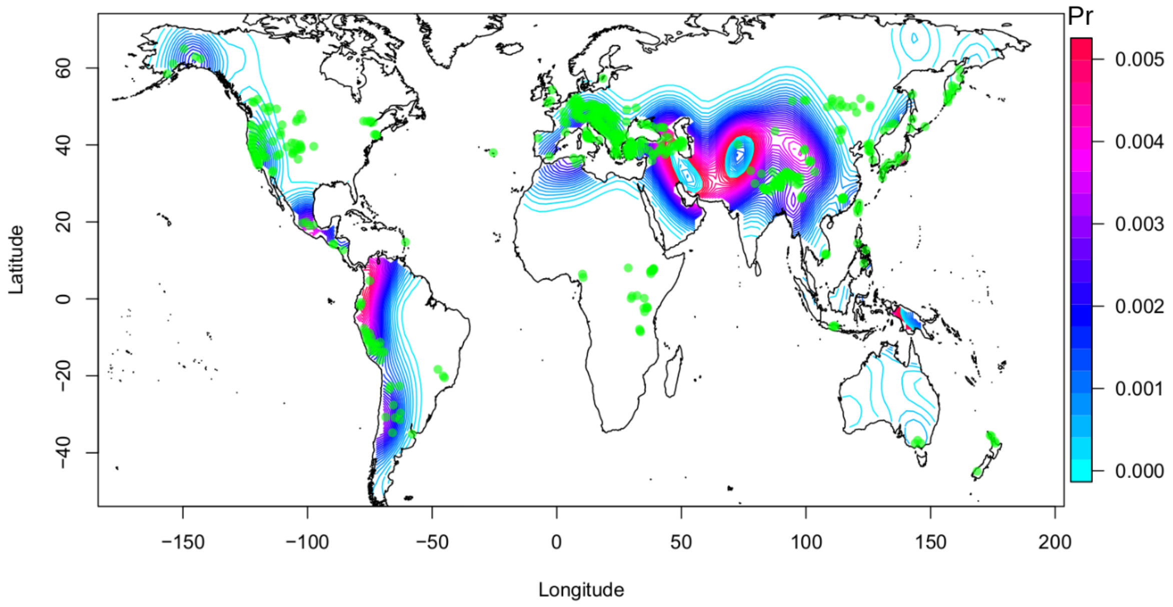

Supplementary Figure 4: Probability of occurrence of compressional tectonic. Green dots are the existing gas discharges listed in this work.

Hexagonal grid

Counting of gas discharges, earthquakes and faults lengths has been obtained by building a discrete global grid that divides the surface of the Earth into equal hexagonal cells. We used the package DggridR for the R programming language. The default "ISEA3H" grid (Icosahedral Snyder Equal Area Aperture 3 Hexagonal Grid) contains 12 pentagonal cells, each having an area exactly 5/6 that of the hexagonal cells, to ensuring that all hexagonal

cells are of equal area. The package allows to select different grid sizes ("resolution"), here we used a resolution from 2 to 9.

Resolution	Cell Area (km^2)	Mean spacing of hexagons center nodes (km)
2	5,667,396	2,540
3	1,889,132	1,480
4	629,711	856
5	209,904	495
6	69,968	287
7	23,323	166
8	7,775	96
9	2,592	56

Supplementary Table 1: Cell area and mean spacing of hexagons center nodes for the ISEA3H grid type. The hexagon radius mentioned in the manuscript is calculated as half of the mean spacing.

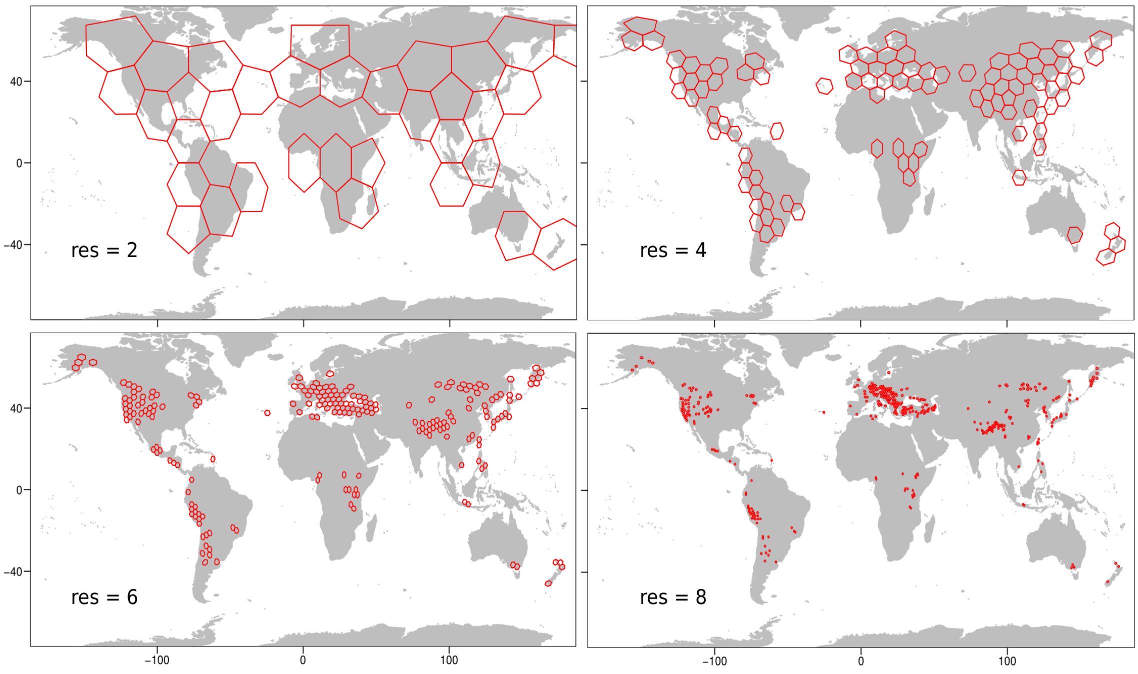

Supplementary Figure 5: Four different ISEA3H hexagonal grid type.

It is worth noting that grid resolutions ≥ 8 are too small to obtain statistically significant counts and resolution 2 (Supplementary Fig. 5) may incorporate gas discharges

belonging to different geodynamic contexts. However we feel that they represent the two end-members of our calculations and, hence, must be taken into account.

Scatterplot between count data

For each resolution, 500 grids have been generated by randomizing the position of the central node. Thus, the number of gas discharges, earthquakes and factive faults lengths have been measured within each hexagon of the each grid. Finally, a spearmen correlation coefficient and a p-value has been calculated between gas discharges counts VS the other count variables. An example for one single grid is shown in Supplementary Fig. 6 . A grid of resolution 2 (1 of 500) is shown on the left with the identification number (ID) for each hexagon. The resulting scatterplot between gas discharges counts and extensional earthquakes counts is shown on the right with the ID of the hexagon in which counts have been measured. The spearman correlation coefficient for this grid and for these variables is 0.697 and it is summarized in the boxplot of Fig. 3 (NF earthquakes).

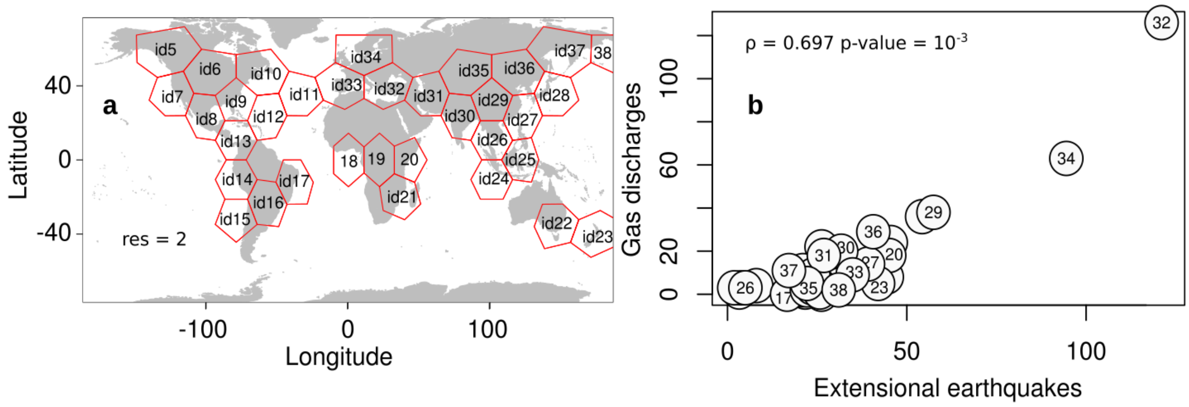

Supplementary Figure 6: (a) Hexagonal grid of resolution 2 and (b) corresponding scatterplot between the counts of gas discharges and extensional earthquakes measured in each hexagonal cell.

Supplementary References

1. Kirkham, R. V., Chorlton, L. B., & Carriere, J. J. Generalized geological map of the world and linked databases, Geological Survey of Canada Open-File, Report 2915d (1995).
2. Barnes, I., Irwin, P.W. & White, D.E. *Global distribution of carbon dioxide discharges, and major zones of seismicity. Water Resour. Invest.* WRI 78-39. U.S. Geol. Surv., Washington DC (1978).

3. Marinov, B. N. & Mirzod, K. H. Northern Afghanistan mineral springs: Bulletin of the Supreme Educational Institution for Geology and Prospecting, 12, 94-100 (1969).
4. Koltov, B. A. et al. Minor elements in carbonated waters of central Afghanistan. *DoM. Akad. Nauk SSSR*, 237, 1486- 1489 (1977).
5. Guigue, S. & Betier, G. Thermal-mineral springs of Algeria: Internat. Assoc. of Scientific Hydrology, General meeting, Oslo, 3, 117-120 (1951).
6. Corti, H. & Camps, J. A to the study of the waters of the Republic of Argentina. Ministry of Agriculture of the Nation, Director General of Mines, Geology, and Hydrology Publication 84, 400 (1930).
7. Tageeva, N. V. Mineral waters of Dzbermuk (ISTISU) in Armenia, in Laboratory for Hydrogeologic Problems Proc. Acad . Sci., U.S.S.R., 1, 212-220 (1948).
8. McLaughlin, R. J. W. & Macumber, J. J . Mineral springs of the Daylesford District. Royal Soc. Victoria Proc., New Series, 81, 143-148 (1968).
9. Conrad, V., Diem, K., Knett, J., Meyer, H. H. & Stockmayer, S. Austrian Bath Book. Ministry of Public Health, State Ministry for Social Administration, Vienna, 330 (1928).
10. Deetjen, P. Drink cures, in Slezak, Paul, ed., Austrian Health Baths and Health Resort Book. Vienna, Bohmann, 125-130 (1975).
11. Kashkai, M. A., Gadzhiev, S. M. & Salmanov, M. A. Biogeochemical characteristics of mineral waters of the northeastern slopes of the greater Caucasus. Acad. Sci. Trans., S.S.R., Geochemistry, 25, 66-70 (1969).
12. Kashkai, M. A., Aliev, V. E. & Mamedov, A. I. Mineral spring fields in the Tutkun Kelbadzharskogo Region of the Azerbaidzhanskoi, S.S.R.. Acad. Sci. Trans., Azerbaidjan S.S.R., 2, 3-21 (1962).
13. Carvalho, A. H. Analytical studies of hot waters. Director General of Mines and Geologic Serv- ices, Portugal, Lisbon, 175 (1955).
14. Graulich, J. M. Mineral and thermal waters of Belgium, in Proc. Symposium II, Mineral and thermal waters of the world. A. Europe. Internat. Geol. Cong. 23d, Czechos- lovakia, 1968, 17- 29 (1969).
15. Josipovic, J. Mineral, thermal, and thermomineral waters in the territory of Bosnia and Hercegovina. Geol. Bull. (Saraijevo), 15, 233-277 (1971).
16. Stancil, B., Stefanovic, M. & Dordevic, D. A contribu- tion to the knowledge of mineral waters of northeastern Bosnia. Geologic Bull., Geologic Institute of Sarajevo, 11, 467-470 (1966).
17. Gonsalves, A. D. Mineral waters of Brazil. Ministry of Agriculture, Directory of Production Statistics, Section of Statistics of Extractive Products (1936).
18. Antonov, K. H. R. & Stoyanov, I. Z. General Hydrogeology of Mineral Water. Sofia, Technika Publishers (1959).

19. Jarocka, A. Mineral and thermal springs of Bulgaria, in Wiktor, Z., ed., Problems of fluoride in balneology. Materials of a Scientific Session, Wroclaw Scientific Society, 23-30 (1968).
20. Petrov, P. Basic regularities in the occurrence of mineral waters in Bulgaria. Works on the geology of Bulgaria series. Engineering Geology and Hydrology, 3, 83-158 (1964).
21. Petrov, P., Martinov, S. B., Limonadov, K. & Straka, Y. Hydrogeology of the baths and mineral waters of Bulgaria. Sofia, Technika Publishers (1970).
22. Spiriev, B. Thermo mineral springs of central Bulgaria related to the structures and tectonic dislocations. *Review Bulgarian Geol. Society,* **21,** 8-32 (1960).
23. Straub, J. The chemical composition of medicinal waters (mineral waters) of Transylvania. Their more dilute components and their biochemical importance. Hungarian Geol. Institute Yearbook, 39, 1-110 (1950).
24. Tanyileke, G. Z., Kusakabe, M. & Evans, W. C. Chemical and isotopic characteristics of fluids along the cameroon volcanic line, Cameroon. *J. African Earth Sci.* **22,** 433–441 (1996).
25. Souther, J. G. Geothermal potential of western Canada. 2nd United Nations Symposium on Development and Use of Geothermal Resources Proc., San Francisco, 1975, 25-267 (1976).
26. Souther, J. G. & Halstead, E. C., Mineral and thermal waters of Canada, in Proc. Symposium II, Mineral and thermal waters of the world. B. Oversea countries. Internat. Geol. Cong., 23d, Czechoslovakia, 1968, 225-256 (1969)
27. Shi, H. A note on carbon-dioxide discharging zones and their seismicities in China, *Seismol. Geol.* **1,** 86-93 (1979).
28. Fetzer, W. G. Mineral springs and deposits of calcium carbonate of Santa Rosa de Cabal (Rio San Ramon). Compilation of official geologic studies in Columbia, National Geologic Service, 6, 433-454 (1945).
29. Mihalic, S. & Trauner, L. Mineral waters of Croatia. Annals of the Balneological Institute, People's Republic of Croatia, 1, 59-133 (1958).
30. Barnes, I. & O'Neil, J. R. Metamorphic reactions in flysch rocks, in Cadek, J ., and Paces, T., eds., Internat. Symposium on Water-Rock Interaction Proc., Czechoslovakia, 1974. Prague, Geological Survey, 30-316 (1976)
31. Franko, O., Gazda, S. & Michalicek, M. Origin and classification of mineral waters of the Western Carpathians. Geologic Institute Dionyza Stura, Bratislava (1975).
32. Kacura, G., Franko, O., Gazda, S & Silar, J. Thermal and mineral waters of Czechoslovakia, in Proc. Symposium II, Mineral and thermal waters of the world. A. Europe. Internat. Geol. Cong., 23d, Czechoslovakia, 1968, 17-29 (1969).

33. De Grys, A., Vera, J. & Goossens, P. A note on the hot springs of Ecuador, in United Nations Symposium on the Development and Utilization of Geothermal Resources Proc. Pisa, 1970. Geothermics Spec. 2, 140-1404 (1970).
34. United Nations Development Programme, Geology, geochemistry, and hydrology of hot springs of the East African rift system within Ethiopia, of Investigation of geothermal resources for power development. Technical report, U.N. Development Program, New York (1973).
35. Godebo, T. Geochemical and isotopic composition of natural waters in the central main ethiopian rift. emphasis on the study of source and genesis of fluoride. PhD thesis Università degli Studi di Ferrara, Italy (2008).
36. Michard, G., Stettler, A., Fouillac, C., Ouzounian, G. & Mandeville, D. Subsuperficial changes in chemical composition of the thermomineral waters of Vichy Basin. Geothermal implications. *Geochem. Jour.* **10,** 15-161 (1976).
37. Risler, J. J. Chemical and isotopic characteristics of the gas of some thermomineral springs of the Central French Massif. Internat. Soc. Hydrothermal Technology, 13th Internat. Cong. Proc., Liege, Belgium (1978).
38. Fricke, K., and Michel, G.,, Mineral and thermal water of the Federal Republic of Germany, in Proc. Symposium II, Mineral and thermal waters of the world. A. Europe. Internat. Geol. Cong., 23d, Czechoslovakia, 1968, 31-57 (1969).
39. Zieschang, J. The mineral water of the German Democratic Republic, in Proc. Symposium 11, Mineral and thermal waters of the world. A. Europe. Internat. Geol. Cong., 23rd, Czechoslovakia, 1968, 59-68 (1969).
40. Dominco, E. & Papastamatoki, A. Characteristics of Greek geothermal waters, in 2d United Nations Symposium on Development and Use of Geothermal Resources Proc. San Francisco, 1975, 109-121 (1976).
41. Pertessis, M. L. Greek hot springs. Greek Geol. Survey Paper. Stability of chemical compositions and temperatures of Greek hot springs. Academy of Athens Proc., 26, 26-38 (1937).
42. Nagy, Z., et al. Chemical analyses of the Hajdùszoboszlò thermal waters. *Hydrological Jour.* **40,** 300-303 (1960).
43. Papp, F. The medicinal waters of Hungary. Internat. Assoc. Of Scientifc Hydrology, General Meeting, Oslo, 3, 154-167 (1948).
44. Zyka, V. Geochemical zonation of mineral waters of central Europe. Geologic Memoirs, Slovak Academy of Science, 9, 26-299 (1958).
45. Chaterji, G. C. Mineral and thermal waters of India, in Proc. Symposium II, Mineral and thermal waters of the world. B. Oversea countries. Internat. Geol. Cong., 23rd, Czechoslovakia, 1968, 21-43 (1969).

46. Shankar, R. et al. Geothermal exploration of the Puga and Chumathang geothermal fields, Ladakh, India, in 2d United Nations Symposium on Development and Use of Geothermal Resources Proc., San Francisco, 1975, 24-258 (1976).
47. Krishnaswamy, V. S. A review of Indian geothermal provinces and their potential for energy utilization, in 2d United Nations Symposium on Development and Use of Geothermal Resources Proc., San Francisco, 1975, 143-156 (1976).
48. Waring, G. A. Thermal springs of the United States and other countries of the world. a summary. Revised by Reginald R. Blankenship and Ray Bentall (1965). U.S. Geol. Survey Prof. Paper 492 (1883).
49. Baldi, P., Ferrara, G. C., Masselli, L., and Pieretti, G. Hydrogeochemistry of the region between Monte Amiata and Rome. *Geothermics* **2,** 124-141 (1973).
50. Damiani, A. V. & Moretti, A. Italian thermal and mineral springs. Proc. Symposium 11, Mineral and thermal waters of the world. A. Europe, Internat. Geol. Cong., 23d, Czechoslovakia 1968, 87-98 (1969).
51. Panichi, C. & Tongiorgi, E. Carbon isotopic composition of CO_2 from springs, fumaroles, mofettes, and travertines of central and southern Italy. Preliminary prospection method of geothermal area. 2nd United Nations Symposium on Development and Use of Geothermal Resources Proc., San Francisco 1975, 815-825 (1976).
52. Chiodini, G., Valenza, M., Cardellini, C. & Frigeri, A. A New Web-Based Catalog of Earth Degassing Sites in Italy. *Eos Trans AGU* **89**, 341 (2008).
53. Cardellini, C., Chiodini, G., Frigeri, A., Bagnato, E., Aiuppa, A. & McCormick, B. The development of a new database of gas emissions: MAGA, a collaborative web environment for collecting data. American Geophysical Union, Fall Meeting 2013, V31B-2708 (2013).
54. Sumi, K. Distribution map of hot springs in Japan (2nd edition). Geological Survey of Japan. Scale 1:2,000,000 (1975).
55. Walsh, J. Mineral and thermal waters of Kenya. Proc. Symposium II, Mineral and thermal waters of the world. B. Oversea countries, Internat. Geol. Cong., 23d, Czechoslovakia 1968, 10-110 (1969).
56. Conny, G., Demians D'Archimbaud, J. & Surcin, J. Geothermal prospecting in the French Antilles. United Nations Symposium on the Development and Utilization of Geothermal Resources Proc., Pisa, Geothermics Spec. Issue 2, 57-72 (1970).
57. Duzelkovski, D. & Strackov, M. A brief review of the thermal and mineral waters of the Socialist Republic of Macedonia and their relation to the tectonic structure. Technology, 1260-1267 (1973).
58. Blasquez L. The Geysers, solfataras, and springs of the Sierra de San Andres, Michoacan. UNAM, Institute of Geology Bull. 61, 1-46 (1961).

59. Ignacio Villalobos, C. Preliminary hydrochemical study of some thermal and medicinal springs of the Republic of Mexico. *Geol. Metallurgy* **3,** 99-114 (1967).
60. Mocina, B. & Banwell, C. J. Chemical studies in Mexican geothermal fields. United Nations Symposium on the Development and Utilization of Geothermal Resources Proc., Pisa, Geothermics Spec. Issue 2, 1377-1391 (1970).
61. Girault, F. et al. Large-scale organization of carbon dioxide discharge in the Nepal Himalayas. *Geophys. Res. Lett*. **41,** 6358–6366 (2014).
62. New Zealand Chemistry Division, Department of Scientific and Industrial Research files.
63. Fomichev, M. M. Mineral waters of North Korea. Bull. Moscow Assoc. for the Study of Nature, Geol. Section 35, 125-130 (1960).
63. Fomichev, M. M. The Chokrak hydrogen sulfides prings. Laboratory for Hydrogeologic Problems Proc., Acad. Sci., U.S.S.R. 1, 212-220 (1948).
64. Komada, I. The cold carbon dioxated spring of Shoseiri. Geol. Survey of Chosen (Korea) 7, 33-38 (1925).
65. Heming, R. F. The mineral and thermal waters of the Territory of Papua and New Guinea. Proc. Symposium 11, Mineral and thermal waters of the world. A. Europe, Internat. Geol. Cong., 23d, Czechoslovakia 1968**,** 293-304 (1969).
66. Alva Saldana, L. Chemical analyses of some mineral waters of Ancash. *Bull. Chem. Soc. Perù* **3,** 76-84 (1937).
67. Carcamo Marquez, V. Chemical analyses oft hirty mineral waters of Perù. *Bull. Chern. Soc. Perù* **3,** 15-45 (1937).
68. Ramos, I. Thermalisms in Perù. *Bull. National School of Engineering* **16,** 3-97 (1943).
69. Zapata Valle, R. Aguas minerales del Perù. Estud. Espec. Serv. de Geol. y Mineria del Peru, Lima 2 (1973).
70. Feliciano, J. M. A study of thermal springs in the Philippines. Proc. 3d Pan-Pacific Science Congress, Tokyo 1, 804-811 (1928).
71. Dowgiallo, J., Plochniewski, Z. & Szpakiewicz, M. Map of the mineral waters of Poland. Geologic Institute, Dept. of Geologic Science, Polish Acad. Sci., 11 (1974).
72. Fistek, J. Some notes on the occurrence and origin of carbonated mineral water of the Klodzkiej Basin. *Geol. Rev.* **19,** 192-195 (1971).
73. Marques, J. M., Carreira, P. M. Questions and answers ascribed to Chaves CO_2-rich thermal waters conceptual model (N Portugal): A Review. *Procedia Earth Planet. Sci.* **17,** 654–657 (2017).
74. Geaminu, N., Geamanu, V., Lungu, P. & Lazu, I. Manifestations of carbon dioxide in the ground waters of the external Carpathian flysch between the valleys of Tazlaul Mare and Zabala. Geologic Institute for Technical and Economic Studies, Series E, Hydrogeology 9, 135-148 (1971).

75. Ghenea, C. & Nicolescu, M., A general review of the mineral and thermal waters of the world. Proc. Symposium 11, Mineral and thermal waters of the world. A. Europe, Internat. Geol. Cong., 23d, Czechoslovakia 1968**,** 99-112 (1969).
76. Mihaila, N. Hydrogeologic and hydrochemical researches in the area covered by the Oradea and Aled Sheets. Geologic Institute for Technical and Economic Studies, Series E, Hydrogeology 9, 104-138 (1971).
77. Kamenskiy, I. L. et al. Components of the upper mantle in the volcanic gases of Kamchatka (according to He, Ne, Ar, and C isotopy). *Geochem. Internat.* **13,** 35-48 (1976).
78. Ivanov, V. V., Ovchinnikov, A. M. & Yarotzky, L. A. Map of undergound mineral waters of the U.S.S.R, scale 1:7,500,000. Ministry of Public Health, U.S.S.R. Health Resort and Physiotherapeutics, State Research Institute (published in English). --1960, Map of underground mineral waters of the U.S.S.R. scale 1:7,5000,000, Explanatory notes: Ministry of Public Health, U.S.S.R. Health Resort and Physiotherapeutics, State Research Institute (1960).
79. Orlova, L. M. Mineral springs, Galushko, YA. A., ed., Mineral resources of the Chitinsko region. Acad. Sci., U.S.S.R., 141 (1959).
80. Radojicic, S. & Jarijic, M. A contribution to the knowledge of thermal mineral water of the P.R. of Serbia-Obreovacka Banja. Bull. Engineering Geology and Hydrology, Belgrade, 1, series B, 111-128 (1960).
81. Milojevic, N. A contribution to the knowledge of the thermal mineral waters of the Kosovskometohija Region. Bull. Engineering Geology and Hydrology, Belgrade, 1, series B, 9-109 (1960).
82. Miholic, S. Mineral waters of the Pohorskog Region. Geol. Bull., Zagreb, 1, 111-124 (1947).
83. Cruz-San Julian, J., Garcia-Rossell, L. & Garido-Blasco, J. Thermal waters of the province of Granada. *Bull. Geology and Mining* **83,** 266-275 (1972).
84. Engqvist, P. Mineral and thermal waters of Sweden. Proc. Symposium 11, Mineral and thermal waters of the world. A. Europe, Internat. Geol. Cong., 23d, Czechoslovakia 1968**,** 127-131 (1969).
85. Nussberger, G., Cadisch, J ., Keller, A. & Werder, J. The mineral and health springs of Switzerland. The Swiss Soc. of Analytical Chemistry of the Confederation's Ministry of Health and the Swiss Assoc. for Balneology and Climatology, Bern Zimmermann and Company, 201 (1937).
86. White, D. E. & Truesdell, A. H. Geothermal resources of Taiwanan evaluation. U.S. Geol. Survey project report (IR), TA-I, Taiwan Investigations, 30 (1970).

87. Nzaro, M. A. Geothermal resources of Tanzania. United Nations Symposium on the Development and Utilization of Geothermal Resources Proc., Pisa, Geothermics Spec. Issue 2, 1039-1043 (1970).
88. Walker, B. G. Springs of deep seated origin in Tanzania. Symposium 11, Mineral and thermal waters of the world. B. Oversea countries, Internat. Geol. Cong., 23d, Czechoslovakia 1968**,** 171-180 (1969).
89. Djellouli, A. The thermal and mineral waters of Tunisia. Symposium 11, Mineral and thermal waters of the world. B. Oversea countries, Internat. Geol. Cong., 23d, Czechoslovakia 1968**,** 181-190 (1969).
90. Alpan, S. Geothermal energy explorations in Turkey. 2nd United Nations Symposium on Development and Use of Geothermal Resources Prot., San Francisco 1975, 2-28 (1976).
91. Erent Oz, C. & Temek, Z. Thermal and mineral waters of Turkey. in Proc. Symposium II, Mineral and thermal waters of the world. Symposium 11, Mineral and thermal waters of the world. B. Oversea countries, Internat. Geol. Cong., 23d, Czechoslovakia 1968**,** 75-84 (1969).
92. Kurtman, F. & Samilgil, E. Geothermal energy possibilities, their exploration and evaluation in Turkey. 2nd United Nations Symposium on Development and Use of Geothermal Resources Prot., San Francisco 1975, 447-457 (1976).
93. Dixon, C. G. & Morton, W. H. Thermal and mineral springs in Uganda. Symposium 11, Mineral and thermal waters of the world. B. Oversea countries, Internat. Geol. Cong., 23d, Czechoslovakia 1968**,** 191-200 (1976), Thermal and mineral springs in Uganda, United Nations Symposium on the Development and Utilization of Geothermal Resources Proc., Pisa, Geothermics Spec. Issue 2, 1035-1038 (1970).
94. Edmunds, W. M., Taylor, B. J. & Downing, R. A. Mineral and thermal waters of the United Kingdom, in Proc. Symposium II, Mineral and thermal waters of the world. A. Europe: Internat. Geol. Cong., 23d., Czechoslovakia, 1968, 139-158 (1969).
95. Allen, E. T. & Day, A. L. Hot springs of the Yellowstone National Park: Carnegie Institution of Wash- ington Pub. 466, Baltimore, Waverly Press, Inc., 525 (1985).
96. Berkstresser, C. F. Data for springs in the southern coast, transverse, and peninsular ranges of California. U.S. Geol. Survey open-file rept. (1968).
97. Crook, J. K. The mineral waters of the United States and their therapeutic uses. New York and Philadelphia, Lea Brothers and Company (1899).
98. Feth, J. H. et al. Preliminary map of the conterminous United States showing depth to and quality of shallowest ground water containing more than 1,000 parts per million dissolved solids. U.S. Geol. Survey Hydrologic Atlas HA-199 (1965).
99. George, R. et al., Mineral waters of Colorado. *Colorado Geol. Survey Bull.* **11,** 1-474 (1920).

100. Grantz, A., White, D. E., Whitehead, H. C. & Tagg, A. R. Saline springs, Copper River lowland, Alaska. *Am. Assoc. Petroleum Geologists Bull.* **46,** 1990-2002 (1974).
101. Mariner, R. H., Rapp, J. B., Willey, L. M. & Presser, T. S. The chemical composition and estimated minimum thermal reservoir temperatures of the principal hot springs of northern and central Nevada: U.S. Geol. Survey open-file rept., 27-32 (1974).
102. Milligan, J. H., Marsell, R. E. & Bagley, J. M. Mineralized springs in Utah: Utah Water Research Board Report WG 23-6, 50 (1966).
103. Peale, A. C. Lists and analyses of the mineral springs of the United States. U.S. Geol. Survey Bull. 32, 235 (1886).
104. Richter, D. H., Lamarre, R. A. & Donaldson, D. E. Soda Creek Springs-Metamorphic waters in the eastern Alaska Range. *U.S. Geol. Survey Jour. Research,* **1,** 523-528 (1973).
105. U.S. Geological Survey files.
106. Fontaine, H. Thermomineral water of central Vietnam: *Geologic Archives of Vietnam*, **4,** 35-124 (1957).
107. Gleeson, T., Befus, K. M., Jasechko, S., Luijendijk, E. & Cardenas, M. B. The global volume and distribution of modern groundwater. *Nature Geosci.* **9,** 161–164 (2016).
