# Supplementary material for: Global-scale control of extensional tectonics on CO2 earth degassing
Source: Nat Commun. 2018 Nov 2;9:4608. doi: 10.1038/s41467-018-07087-z (PMC6214998; doi:10.1038/s41467-018-07087-z)
Supplement: Supplementary file 3 — Description of Additional Supplementary Files [file 41467_2018_7087_MOESM3_ESM.pdf]

Description of Additional Supplementary Files

File Name: Supplementary Data 1

Description: Geographic coordinates of the gas discharges. Values are expressed in decimal longitude and latitude. The following additional information is provided: country, altitude (extracted from the global digital elevation model GTOPO30, https://lta.cr.usgs.gov/GTOPO30), geology and era of the terrain (extracted from the general geologic map of the world; https://mrdata.usgs.gov/geology/worldgeol.html), distance from the nearest fault and sliptype, distance from the nearest Holocene volcano.
